# Identification of the first COVID-19 infections in the US using a retrospective analysis

**DOI:** 10.21203/rs.3.rs-707353/v1

**Published:** 2021-07-24

**Authors:** David García-García, Enrique Morales, Cesar de la Fuente-Nunez, Isabel Vigo, Eva S. Fonfría, Cesar Bordehore

**Affiliations:** University of Alicante: Universitat d’Alacant; University of Alicante: Universitat d’Alacant; University of Pennsylvania Department of Bioengineering; University of Alicante: Universitat d’Alacant; University of Alicante: Universitat d’Alacant; University of Alicante: Universitat d’Alacant

**Keywords:** COVID-19, SARS-CoV-2, REMEDID

## Abstract

Accurate detection of early COVID-19 cases is crucial to drastically reduce infection, hospitalization, and death rates. However, it remains a challenge and methods for identifying initial COVID-19 cases are urgently needed. Here, we used the results from a seroprevalence study in 50 US states to apply our Retrospective Methodology to Estimate Daily Infections from Deaths (REMEDID) with the aim of analyzing the initial stages and spread of SARS-CoV-2 infections across the United States (US). Our retrospective data analysis revealed that the virus likely entered the country through California on December 28, 2019, which corresponds to 16 days before the officially recognized entry date established by the CDC. Thus, REMEDID provides evidence that SARS-CoV-2 entered the U.S. earlier than previously reflected in official data. Collectively, our mathematical modeling more accurately estimates the initial COVID-19 cases in the US, may be extrapolated to other countries, and may be used to retrospectively track the progress of the pandemic. Approaches such as REMEDID may enable health authorities to accelerate preventative measures aimed at controlling pandemics within weeks of their onset.

SARS-CoV-2 was detected for the first time in Wuhan, China, in December 2019 (1) subsequently spreading rapidly throughout the world. However, its dissemination may have been even faster than previously appreciated. In the Unites States of America (US), according to data aggregated by USAFacts (accessed on March 15, 2021, from https://usafacts.org/visualizations/coronavirus-covid-19-spread-map/) from the Centers for Disease Control and Prevention (CDC), state- and local-level public health agencies, the first documented cases emerged in Washington state on January 22, 2020, followed by Illinois (on January 24), and California and Arizona (both on January 26). These were isolated cases, since the second/third report of cases in these states only took place 38/39, 7 /37, 1/3, and 40/41 days later. Identifying the very first case of a pandemic is an arduous task, which has been further emphasized in the context of COVID-19 due to the high proportion of asymptomatic and mildly symptomatic individuals (2). Several attempts have been made to this end. In France, a retrospective analysis of respiratory samples of an individual hospitalized on December 27, 2019, was positive for SARS-CoV-2, which is around a month before the first case had been reported (Deslandes et al., 2020). In US, retrospective analysis of blood samples identified virus introduction earlier than reported in Illinois, Massachusetts, Wisconsin, Pennsylvania, and Mississippi (Althoff et al., 2021), and even between December 13–16, 2019, in California, Oregon, and Washington (Baravaraju et al., 2020). In order to provide insights into the early stages of the COVID-19 outbreak in the US, here we perform an independent retrospective data analysis based on reported deaths, clinical information of the illness, and the results of a seroprevalence study (Bajema et al, 2020).

Overall, COVID-19 deaths have been more thoroughly documented than infections. Our Retrospective Methodology to Estimate Daily Infections from Deaths (REMEDID) (3) can be applied if the case fatality ratio (CFR) and the probabilistic distributions of incubation period (IP) and time from illness onset to death (IOD) are known. From initial cases in Wuhan, Linton et al. (4) approximated a lognormal distribution to IP (mean=5.6 days, median=5 days), and IOD (mean=14.5 days, median=13.2 days). The CFR is estimated for each state from a seroprevalence study, which estimate the accumulated infections up to a date close to the realization of the study. The seroprevalence study by Bajema et al. (5) was carried out at the following four different time periods in 2020: July 27 - August 13; August 10 – 17; August 24 - September 10; and September 8 – 24. The accumulated infections detected for each period are associated to a specific date for each state. Although the number of accumulated infections in a given period should be larger than those from any given previous period, this is not always the case when dealing with a relatively low number of cases per time interval. Therefore, for each state, we consider the averaged infections for the four periods in relation to the average data from such time periods. The accumulated deaths up to those dates, plus the proportional deaths detected subsequently according to the convolution of IP and IOP distributions, are used to estimate a mean CFR for each state. Finally, REMEDID was applied to estimate the daily infections occurring in each state. The REMEDID infections time series present some advantages with respect to official records since they are compatible with: (i) the stochastic information available about the COVID-19, such as IP and IOD distributions; (ii) the seroprevalence studies, then providing a realistic total amount of infections; and (iii) daily death time series. When applying the REMEDID, the resultant time series must rounded to integer (positive) numbers. Then, the first non-null element defines the date of the first infection.

Figure 1 and Table 1 show the dates of the first officially documented and REMEDID cases, respectively, for each state. The first REMEDID case in the US was located in California on December 28, 2019, that is 29 days before the first officially documented case, and 3 days before the Wuhan Municipal Health first reported a cluster of pneumonia cases of unknown origin (6). The earliest REMEDID case is 2 weeks later than those retrospectively reported by Baravaraju et al. (2020), meaning that early infections may have been produced in an above-average proportion of individuals with low risk of death. The second state presenting a REMEDID case was Washington, also on the West Coast of the US, and the third was New York. These observations are consistent with the fact that California and New York receive the largest number of flight connections from China. In December 2019, the only two direct flights from Wuhan airport to the US were to San Francisco (8071 passengers) and New York (5849 passengers), while other Chinese airports sent 299,278 passengers to California, 97,897 to New York, 38,149 to Washington state, and 266,273 to other 7 states (data.transportation.gov). Therefore, it makes sense that California had the first case because this was the state that received the most travelers from Wuhan, China. The first and second documented cases in US were a man and a woman travelling from Wuhan to Washington and Illinois states with arrival dates on January 15 and 13, 2020, respectively (7, 8). The case of Illinois did not lead to a local outbreak since it was rapidly isolated. Indeed, apparently only the patient’s husband was infected, accounting for the first documented secondary transmission of COVID-19 in the US. However, the Illinois case was not the only one, since Althoff et al. (2021) retrospectively reported a case on January 7, 2020. It make sense to think that there were more cases since the two earliest documented cases were detected because the hosts presented symptoms and went to the hospital, which happens in a low portion of infections. REMEDID infections allow the study of the early spread of mild and asymptomatic (and undetected) cases, assuming that their proportion was similar at the beginning of the epidemic and during the period covered by the seroprevalence study. Differences are remarkable. For example, Illinois dropped to the 13^th^ position using our REMEDID infection score. On average, the first REMEDID cases occurred 32 days prior to the official case count, revealing that: (i) SARS-CoV-2 spread to the US states a month earlier in average than previously reported in official records; (ii) there was a generalized under-detection of cases during the beginning of the pandemic. Only Arizona and Illinois showed earlier first cases in documented infections than in our REMEDID analysis. Finally, West Virginia was the last state to report a COVID-19 infection (on March 17, 2020), contrary to our REMEDID analysis that identified Wyoming as the last state on its ranking (on February 28, 2020).

These results are important to understand viral spread and provide substantial evidence that COVID-19 transmission occurs more rapidly than previously observed through official recorded data. This is underscored by the observation that SARS-CoV-2 arrived in the US before it was even reported by the Wuhan authorities in China. The situation was similar in Spain where, during the first COVID-19 wave, the 1^st^ official case was detected on February 20, 2020, in contrast to our REMEDID model, which identified the 1^st^ case 43 days earlier on January 8 (3). Our mathematical modeling results reveal a generalized and significant delay in the detection of the first viral cases in the US, which may extend to numerous other countries around the globe.

## Figures and Tables

**Figure 1:**
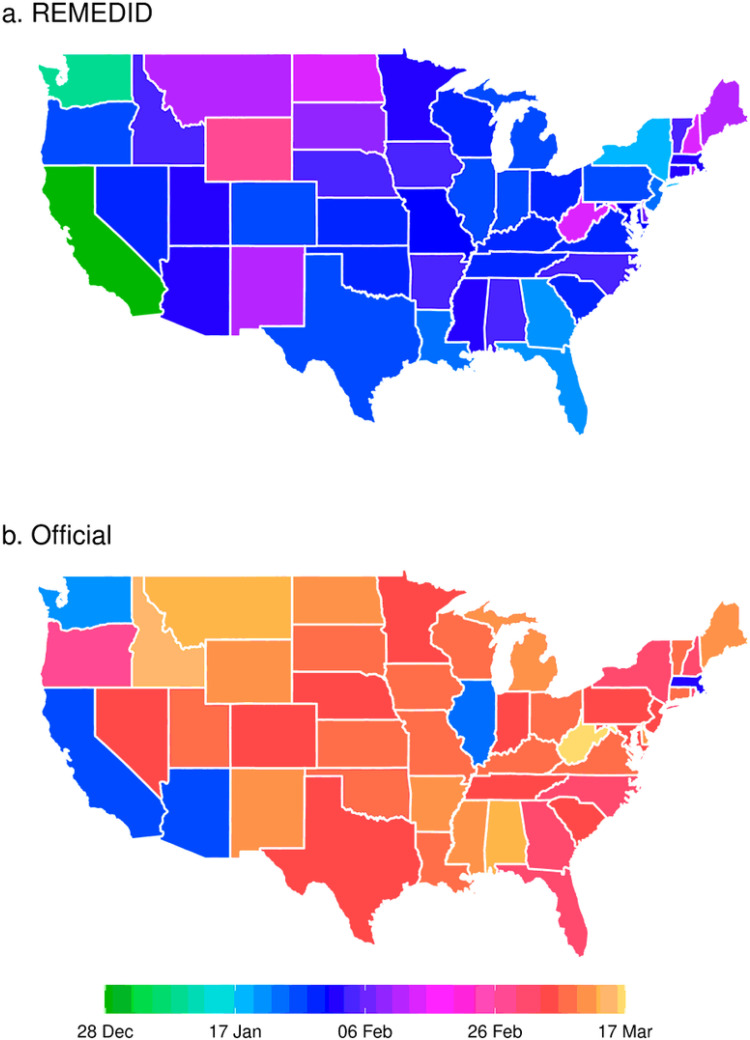
REMEDID modeling predicts that initial COVID-19 cases in the US occurred earlier than previously recorded in official records. First COVID-19 cases recorded for each state for: a) REMEDID infections, and b) officially documented infections. The scale shows December 2019, and subsequent months (January-March) from the year 2020.

**Table 1.** Dates corresponding to the first COVID-19 cases for each state within the US based on both our REMEDID modeling and officially reported data. Positive values under the “Difference in days” column correspond to the difference in number of days between our REMEDID modeling and officially recorded records. A positive value means that the first estimated REMEDID case was ahead of that recorded in official records and a negative value signifies the reverse.

State	Date of 1^st^ REMEDID case	Date of 1^st^ documented case	Difference in days
Alabama, AL	3/2/20	13/3/20	39
Alaska, AK	10/2/20	12/3/20	31
Arizona, AZ	2/2/20	26/1/20	−7
Arkansas, AR	3/2/20	11/3/20	37
California, CA	28/12/19	26/1/20	29
Colorado, CO	28/1/20	6/3/20	38
Connecticut, CT	2/2/20	9/3/20	36
Delaware, DE	7/2/20	12/3/20	34
Florida, FL	22/1/20	2/3/20	40
Georgia, GA	22/1/20	3/3/20	41
Hawaii, HI	12/2/20	7/3/20	24
Idaho, ID	3/2/20	14/3/20	40
Illinois, IL	28/1/20	24/1/20	−4
Indiana, IN	28/1/20	6/3/20	38
Iowa, IA	3/2/20	9/3/20	35
Kansas, KS	30/1/20	8/3/20	38
Kentucky, KY	30/1/20	9/3/20	39
Louisiana, LA	23/1/20	9/3/20	46
Maine, ME	11/2/20	12/3/20	30
Maryland, MD	31/1/20	6/3/20	35
Massachusetts, MA	1/2/20	1/2/20	0
Michigan, MI	27/1/20	10/3/20	43
Minnesota, MN	1/2/20	6/3/20	34
Mississippi, MS	2/2/20	12/3/20	39
Missouri, MO	31/1/20	8/3/20	37
Montana, MT	12/2/20	13/3/20	30
Nebraska, NE	5/2/20	6/3/20	30
Nevada, NV	29/1/20	5/3/20	36
New Hampshire, NH	15/2/20	2/3/20	16
New Jersey, NJ	24/1/20	5/3/20	41
New Mexico, NM	11/2/20	11/3/20	29
New York, NY	19/1/20	2/3/20	43
North Carolina, NC	3/2/20	3/3/20	29
North Dakota, ND	13/2/20	12/3/20	28
Ohio, OH	30/1/20	9/3/20	39
Oklahoma, OK	29/1/20	7/3/20	38
Oregon, OR	26/1/20	29/2/20	34
Pennsylvania, PA	27/1/20	6/3/20	39
Rhode Island, RI	12/2/20	1/3/20	18
South Carolina, SC	30/1/20	6/3/20	36
South Dakota, SD	8/2/20	9/3/20	30
Tennessee, TN	30/1/20	5/3/20	35
Texas, TX	26/1/20	5/3/20	39
Utah, UT	2/2/20	7/3/20	34
Vermont, VT	4/2/20	8/3/20	33
Virginia, VA	29/1/20	8/3/20	39
Washington, WA	9/1/20	22/1/20	13
West Virginia, WV	15/2/20	17/3/20	31
Wisconsin, WI	30/1/20	9/3/20	39
Wyoming, WY	28/2/20	12/3/20	13
